# Adherence to international dietary recommendations in association with all-cause mortality and fatal and non-fatal cardiovascular disease risk: a prospective analysis of UK Biobank participants

**DOI:** 10.1186/s12916-021-02011-7

**Published:** 2021-06-23

**Authors:** Maryam Kebbe, Min Gao, Aurora Perez-Cornago, Susan A. Jebb, Carmen Piernas

**Affiliations:** 1 Nuffield Department of Primary Care Health Sciences, University of Oxford, Radcliffe Observatory Quarter, Woodstock Road, Oxford, OX2 6GG UK; 2 Pennington Biomedical Research Center, Louisiana State University, Baton Rouge, LA USA; 3 School of Public Health, Peking University Health Science Center, Beijing, China; 4 Cancer Epidemiology Unit, Nuffield Department of Population Health, University of Oxford, Oxford, UK

**Keywords:** Cardiovascular diseases, Diet, Mortality, Dietary recommendations, Cohort study

## Abstract

**Background:**

International dietary guidelines aim to reduce risks of all-cause mortality, cardiovascular disease (CVD), and fatal CVD often associated with poor dietary habits. However, most studies have examined associations with individual nutrients, foods, or dietary patterns, as opposed to quantifying the pooled health effects of adherence to international dietary recommendations. We investigated associations between total adherence to the World Health Organization (WHO) dietary recommendations for saturated fats, free sugars, fibre, and fruits and vegetables and all-cause mortality and fatal and non-fatal CVD.

**Methods:**

We included participants from the UK Biobank cohort recruited in 2006–2010, which provided at least two valid 24-h dietary assessments. We defined adherence to dietary recommendations as ≤ 10% saturated fats, ≤ 10% free sugars, ≥ 25 g/day fibre, and ≥ 5 servings of fruits and vegetables/day. Multivariable Cox-proportional hazards models were used to investigate prospective associations with all-cause mortality and fatal and non-fatal CVD. In cross-sectional analyses, multivariable linear regression was used to examine associations with cardiometabolic risk factors.

**Results:**

Among 115,051 participants (39–72 years), only 29.7%, 38.5%, 22.3%, and 9.5% met 0, 1, 2, or 3–4 recommendations, respectively. There was a lower risk of all-cause mortality among participants meeting more dietary recommendations (*P*_trend_ < 0.001), with a significantly lower risk among participants meeting 2: HR 0.91 (95% confidence interval [CI] 0.85–0.97) and 3–4: HR 0.79 (95% CI 0.71–0.88) recommendations. There was no trend with CVD risk, but a significantly lower risk of fatal CVD with 3–4 recommendations: HR 0.78 (95% CI 0.61–0.98). Meeting more recommendations resulted in significant cross-sectional trends (*P*_trend_ < 0.001) towards lower body fat, waist circumference, LDL cholesterol, apolipoprotein B, triglycerides, alkaline phosphatase, gamma glutammyltransferase, and hs-CRP, but higher glucose and aspartate aminotransferase.

**Conclusions:**

Meeting dietary recommendations is associated with additive reductions in premature mortality. Motivating and supporting people to adhere to dietary guidelines may help extend years of healthy life expectancy.

**Supplementary Information:**

The online version contains supplementary material available at 10.1186/s12916-021-02011-7.

## Background

Current diets are associated with a high burden of non-communicable diseases (NCDs). In 2017, approximately 11 million deaths and 255 million disability-adjusted life years globally were attributable to dietary risk factors [1]. International dietary guidelines aim to reduce the risk of NCDs, including heart disease, stroke, diabetes, and cancer. The World Health Organization (WHO) recommends limiting the intake of saturated fats and free sugars to less than 10% of total energy intake, while increasing intakes of fibre to a minimum of 25 g and fruits and vegetables to five portions per day among other dietary recommendations [2]. With small differences, these recommendations are similar to most national dietary guidelines [3, 4]. However, despite decades of global efforts to align dietary behaviours with these recommendations, a poor diet remains one of the leading risk factors for ill-health [1].

Previous research has shown that diets high in cereals, fruits, vegetables, and fibre and low in free sugars and processed meats are associated with cardiometabolic health benefits [5] and lower mortality [1]. Individually, there is strong evidence that free sugars are associated with increased risk of weight gain [6], while saturated fats increase total blood cholesterol and low-density lipoprotein (LDL) cholesterol concentrations, which may increase the risk of cardiovascular disease (CVD) [7]. Conversely, increased consumption of dietary fibre could help to control blood glucose and lower serum cholesterol concentrations, potentially lowering the risk of CVD [8].

While analyses of diet have frequently investigated associations between individual nutrients or foods and specific disease endpoints, there is limited evidence quantifying the pooled health effects of adherence to international dietary recommendations on all-cause mortality, total and fatal CVD, and cardiometabolic risk factors, especially from large-scale cohort studies. Using data from the UK Biobank study, we aimed to describe adherence to four main WHO dietary recommendations commonly associated with NCDs, investigate the prospective associations between overall adherence to dietary recommendations and all-cause mortality and the risk of total and fatal CVD, and explore whether cross-sectional associations with key cardiometabolic risk factors may explain any observed associations with outcomes.

## Methods

### Study design and participants

The UK Biobank is a national prospective cohort involving a sample of 502,536 participants aged 37–73 years who were recruited between 2006 and 2010. Sociodemographic information, lifestyle factors, medical history, physical and functional measures, and biological samples were collected from participants at baseline or follow-up assessments across 22 assessment centres in England, Scotland, or Wales [9].

### Study measures

#### Sociodemographic, physical, and biological assessments

Face-to-face interviews and self-administered, touchscreen questionnaires conducted at the baseline assessment centre were used to collect information on age, sex, ethnicity, education, smoking status, alcohol consumption, and menopause status for women. Townsend area deprivation index was derived from participant postal codes of residence using aggregated data on unemployment, car and home ownership, and household overcrowding [10]. Physical activity levels over a typical week were self-reported using the validated international physical activity questionnaire, from which the total metabolic equivalent of task (MET) hours per week were calculated [11]. Trained staff took a series of physical measurements, including height, weight, and blood pressure. Additional File 1: Supplementary Table S1 contains details on the collection and analysis of biomarkers of interest, including markers of adiposity and central adiposity, glucose and lipid metabolism, blood pressure, liver enzymatic activity, and inflammation.

#### Dietary assessment

Dietary data were derived from the Oxford WebQ (to estimate average daily intakes of saturated fats, free sugars, and fibre) and a brief touchscreen questionnaire (to estimate daily servings of fruits and vegetables). All UK Biobank participants completed the touchscreen questionnaire on a computer at their initial assessment centre visit. We used the frequency of consumption for (i) fruit (fresh or dried) and (ii) vegetables (excluding potatoes), namely never, less than once per week, once per week, 2–4 times per week, 5–6 times per week, or once or more daily.

The Oxford WebQ is a web-based self-administered dietary assessment tool. It collects information on foods and beverages (up to 206 food items and 32 types of drinks commonly consumed in the UK over the previous 24 h) [12]. Between 2009 and 2010, 70,724 participants completed the Oxford WebQ as part of their baseline assessment. Between 2011 and 2012, all participants with valid email addresses (*n* = 331,013) were invited to complete the Oxford WebQ on four separate occasions every 3–4 months, from which approximately 53% of participants (*n*=176,012) contacted by email completed at least one assessment. A total of 211,050 participants completed at least one WebQ assessment [13].

For this study, average daily intakes for nutrients and energy were calculated from at least two WebQ assessments in order to estimate usual intakes. Total energy and nutrient intakes were generated by multiplying the number of portions consumed by the set quantity of each food portion size and its nutrient composition obtained from the UK Nutrient Databank Food Composition Tables from survey year 6 (2012–2013 and 2013–2014) [14–16]. Dietary fibre (non-starch polysaccharides) was calculated using the Englyst method [17].

In order to exclude dietary under/overreporters, individual estimated energy requirements (EERs) were calculated as basal metabolic rates with the use of the Schofield Equation from the 1985 FAO/WHO/UNU Expert Consultation Report on Human Energy Requirements; implausible energy intakes were assessed by taking the ratio of reported energy intake (EI) to EER (EI:EER) [18].

### Exposure ascertainment

The main exposure was adherence to dietary recommendations based on WHO criteria [2, 19]: ≤ 10% saturated fats, ≤ 10% free sugars, ≥ 25 g/day fibre, ≥ 5 servings of fruits and vegetables/day (including fresh fruit intake, raw vegetable intake, and cooked vegetable intake).

### Outcome ascertainment

All-cause mortality, total CVD, and fatal CVD were obtained through hospital admissions and death registries linked to the UK Biobank. Total CVD was defined as a hospital admission or death using ICD-10 (International Classification of Diseases, 10^th^ revision) codes, including coronary heart disease (I20–I25), congestive heart failure or cardiomyopathy (I50, I50.1, 150.9, I11.0, I13.0, I13.2, I42, I43.1), and total stroke (I60–I64). Hospital admission data were available until June 30th, 2020, in England; October 31st, 2016, in Scotland; and February 29th, 2016, in Wales. Death registries included date of deaths if occurred before July 30th, 2020, in England, Wales, and Scotland.

We selected available biomarkers of the metabolic syndrome, including adiposity and central adiposity (body mass index [BMI], total body fat percentage, waist circumference), glucose metabolism (fasting blood glucose, glycated haemoglobin), lipid metabolism biomarkers (blood lipid fractions [triglycerides, LDL cholesterol, high-density lipoprotein cholesterol, lipoprotein A, and apolipoprotein B]), blood pressure (systolic and diastolic), liver and other organ enzymes (alkaline phosphatase, alanine aminotransferase, aspartate aminotransferase, and gamma glutamyltransferase), and inflammation (high-sensitivity C-reactive protein [hsCRP]).

### Statistical analyses

#### Prospective associations between adherence to dietary recommendations and all-cause mortality, total CVD, and fatal CVD

The primary outcome analysis was based on the number of dietary recommendations met out of a total of 4, using 0 as the reference category. In secondary analyses, we examined associations based on adherence to individual dietary recommendations by mutual adjustment.

We examined longitudinal associations between adherence to dietary recommendations and all-cause mortality, total CVD, and fatal CVD. We used multivariable Cox proportional hazards models with age as the underlying timescale to estimate hazard ratios (HRs) with 95% confidence intervals (CIs) for each analysis of categorical and individual recommendations met. We also used the floated absolute risk method, which relies on group-specific variances to calculate CIs around the estimate of risk in each group (including the reference) and allows comparisons across exposure groups [20]. The proportional hazards assumption was based on Schoenfeld residuals and was not violated for the variables of interest in the adjusted model (P > 0.05). To calculate the time to follow-up, we used age at completion of the last dietary assessment as the start date until age of occurrence of the first event (death or CVD) or censoring date, whichever came first. This analysis was stratified by sex, adjusted for ethnicity (whites, others, unknown), region (England, Scotland, Wales), Townsend index of deprivation (quintiles 1–5 or unknown, with lower scores representing greater affluence), education group (vocational qualifications [NVQ, HND, HNC], any school degree [A-level, AS-level, O-level, GCSE, CSE], higher degree [college, university, of professional degree/qualification], none of the above, unknown), smoking status (never, previous, current, unknown), physical activity (continuous, total MET-hours/week), alcohol consumption (none, occasional < 1 unit/week, moderate 1–14 units/week, heavy > 14 units/week, unknown), menopausal status (yes, no, not applicable [men]), and log-transformed total daily energy.

Sensitivity analyses were conducted for the prospective analysis to exclude participants who had a CVD event within 2 years after completing their last 24-h online dietary assessment to account for reverse causality. A post hoc sensitivity analysis was conducted including people who completed 3+, 4+, and 5+ dietary questionnaires as more dietary questionnaires would reflect usual intakes more accurately. A post hoc exploratory subgroup analysis was also conducted by sex, age at recruitment (< 60 years or ≥ 60 years), smoking status, BMI group, and presence of risk factors (hypertension, diabetes, and high cholesterol).

#### Cross-sectional associations between adherence to dietary recommendations and cardiometabolic markers

We used multivariable linear regression to estimate geometric means (95% CIs) of metabolic syndrome biomarkers according to total adherence to dietary recommendations, adjusted for age, sex, and the same covariates specified above. This analysis included a sub-sample of participants who had cardiometabolic biomarkers measured at baseline, in addition to at least 2 dietary assessments (the first one at baseline and the subsequent ones during the follow-up).

All statistical analyses were performed using Stata (version 14.0; StataCorp LP) statistical software; a two-sided p value was set at < 0.05 for statistical significance.

## Results

A total of 115,051 participants were included in these analyses. Participants were excluded if they met any of the following: did not provide any dietary intake data (*n* = 291,492), CVD prior to baseline assessment or before completing the last dietary assessment (*n* = 47,409), without two or more WebQs (*n* = 1635), pregnancy at baseline (*n* = 175), implausible energy intakes (underreporters, *n* = 1987, overreporters, *n* = 224), BMI < 18.5 kg/m^2^ (*n* = 5347), missing data fruit and vegetable intakes (*n* = 951), and missing physical activity (*n* = 1961) (Additional File 1: Supplementary Figure S1).

### Adherence to dietary recommendations

Nearly one third (29.7%) of participants did not meet any dietary recommendations, while 38.5% of participants met one recommendation, 22.3% met two recommendations, and 9.5% met three or four recommendations (Table 1). Of those meeting only one recommendation, 50.4% met recommendations for free sugars, 25.5% for saturated fats, 18.6% for fruits and vegetables, and 5.5% for fibre (Table 1). A higher number of recommendations were met by women, ethnicities other than White, residence in more affluent areas, and those with higher education. Greater adherence to dietary recommendations was more likely to occur in those reporting other healthy behaviours, including higher levels of physical activity and lower alcohol consumption, as well as those with a healthy weight, healthy blood cholesterol, and a diagnosis of hypertension or diabetes.

**Table 1:** Total adherence to international dietary recommendations by the World Health Organization (WHO) (*n* = 115,051)

		% meeting recommendations (WHO)			
	Total ***n*** = 115,051	Zero ***n*** = 36,514	One ***n*** = 44,989	Two ***n*** = 23,835	Three/four ***n*** = 9713
**Dietary recommendations**		29.7	38.5	22.3	9.5
% Meeting saturated fat	28.4	0	25.5	55.2	83.1
% Meeting fibre	10.9	0	5.5	18.8	57.4
% Meeting free sugars	41.9	0	50.4	71.2	87.7
% Meeting fruit and vegetables	26.1	0	18.6	54.8	88.8
**Sociodemographic characteristics**					
**Age, mean (SD)**	55.8 (7.8)	55.4 (8.0)	55.7 (7.8)	56.3 (7.6)	56.7 (7.5)
**Sex (%)**					
Female	57.0	27.3	38.3	23.8	10.5
Male	43.0	32.9	38.7	20.2	8.2
**Ethnicity (%)**					
Whites	96.6	29.9	38.6	22.1	9.4
Others	3.4	24.3	36.5	26.9	12.4
**Region (%)**					
Scotland	5.3	31.8	39.6	20.5	8.1
Wales	3.1	29.5	37.5	22.6	10.4
England	91.6	29.6	38.5	22.3	9.6
**Townsend index (quintiles)**					
Q1	20.1	30.3	38.7	21.9	9.2
Q3	20.2	30.2	38.3	22.1	9.4
Q5	19.0	28.5	38.5	22.7	10.3
**Education group (%)**					
Vocational qualification (NVQ, HND, or HNC)	12.4	31.5	38.7	21.2	8.7
Any school degree (A-level, AS-level, O-level, GCSE, CSE)	29.3	30.8	38.9	21.5	8.8
Higher degree (college, university or professional degree/qualification)	52.2	28.8	38.3	22.9	10.0
None of the above	5.8	29.1	38.0	22.5	10.4
**Behavioural risk factors**					
**Alcohol (%)**					
Occasional (< 1 unit/week)	2.4	34.4	34.5	20.4	10.7
Moderate (1–14 units/week)	34.7	31.0	37.5	21.9	9.6
Heavy (> 14 units/week)	45.1	27.5	40.7	23.0	8.8
None	2.4	33.8	34.5	20.7	11.0
**Smoking status (%)**					
Never	58.0	30.8	38.2	21.8	9.2
Previous	35.1	26.9	38.5	23.9	10.7
Current	6.8	35.2	40.9	17.8	6.0
**Physical activity (MET hours/week), mean (SD)**	41.0 (50.3)	37.2 (48.2)	39.8 (49.3)	44.3 (50.8)	50.7 (57.1)
**Total energy intake (kcal/day), mean (SD)**	2070 (510)	2129 (473)	2051 (500)	2012 (541)	2092 (562)
**Medical conditions**					
**Body mass index group (%)**					
Healthy weight (18.5 to < 24.9 kg/m^2^)	40.4	29.6	37.9	22.1	10.4
Overweight (25 to < 29.9 kg/m^2^)	40.8	30.1	38.7	22.2	8.9
Obesity I (30–34.9 kg/m^2^)	13.8	29.3	39.2	22.9	8.7
Obesity II (≥ 35 kg/m^2^)	4.9	29.0	39.5	22.3	8.9
**Hypertension (%)**					
Yes	47.2	28.9	38.6	22.7	9.8
**Diabetes (%)**					
Yes	3.8	20.5	37.8	28.4	13.4
**High cholesterol (%)**					
Yes	82.3	29.9	38.7	22.2	9.3

### Associations between adherence to dietary recommendations and all-cause mortality as well as cardiovascular disease incidence and mortality

**Fig. 1:**
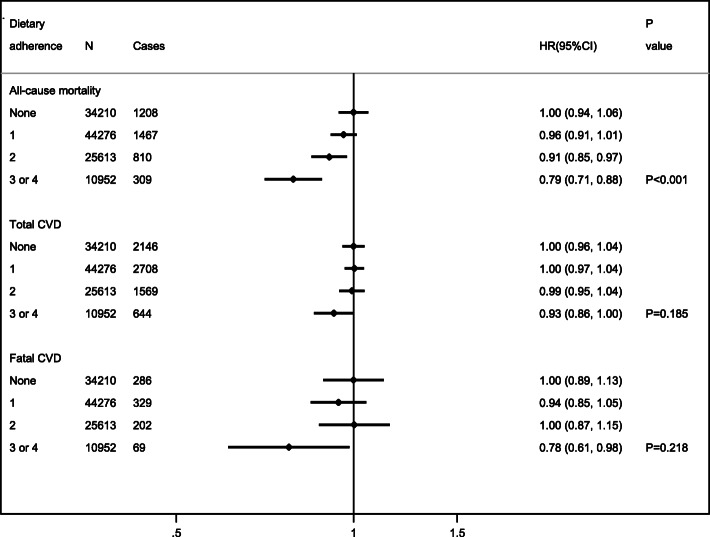
Associations between dietary adherence and all-cause mortality, total CVD, and fatal CVD risks (*n* = 115,051). Abbreviations: CVD (cardiovascular disease), HR (hazard ratio), and CI (confidence intervals). Adjusted HRs, 95%CI, and p values were estimated through multivariable Cox-proportional hazards models. Models included age as the underlying timescale, were stratified by sex, and adjusted for ethnicity (Whites, others, unknown), region (England, Scotland, Wales), Townsend index of deprivation (quintiles 1–5 or unknown, with lower scores representing greater affluence), education group (vocational qualifications [NVQ, HND, HNC], any school degree [A-level, AS-level, O-level, GCSE, CSE], higher degree [college, university, of professional degree/qualification], none of the above, unknown), smoking status (never, previous, current, unknown), physical activity (continuous, total MET-hours/week), alcohol consumption (none, occasional < 1 unit/week, moderate 1–14 units/week, heavy > 14 units/week, unknown), menopausal status (yes, no, not applicable [men]), and log-transformed total daily energy

On average, the follow-up time from baseline was 10.6 years for total CVD and 11.2 years for fatal CVD and all-cause mortality. Increasing adherence to dietary recommendations was associated with lower all-cause mortality (HR 0.96, 95% CI 0.91–1.01 [one recommendation met]; HR 0.91, 95% CI 0.85–0.97 [two recommendations met]; HR 0.79, 95% CI 0.71–0.88 [three or four recommendations met]; *P*_trend_ < 0.001). There was no significant trend in the association with CVD incidence or fatal CVD, though the latter was significantly lower if 3 or 4 recommendations were met (HR 0.78, 95% CI 0.61–0.98) (Fig. 1). The observed associations were not generally explained by one single recommendation, except for a significant association between adherence to ≥ 5 portions of fruits and vegetables a day and lower all-cause mortality (HR 0.91, 95% CI 0.85–0.98) (Additional File 1: Supplementary Figure S2).

In a sensitivity analysis excluding participants who had a CVD event within 2 years of completing their last 24-h online dietary assessment, the associations between total adherence to dietary recommendations and the risk of all-cause mortality, total CVD, and fatal CVD events were unchanged (Additional File 1: Supplementary Table S2). A second sensitivity analysis among participants who completed more dietary questionnaires generally showed consistent associations, especially among people completing 3+ and 4+ dietary questionnaires, although precision was reduced in most cases due to smaller samples (Additional File 1: Supplementary Table S3).

A post hoc subgroup analysis generally showed very similar patterns of associations with all the outcomes across groups of age, sex, BMI, smoking, and presence of risk factors, although a number of associations lacked adequate precision due to smaller samples (Additional File 1: Supplementary Table S4).

### Associations between adherence to dietary recommendations and cardiometabolic markers

**Fig. 2:**
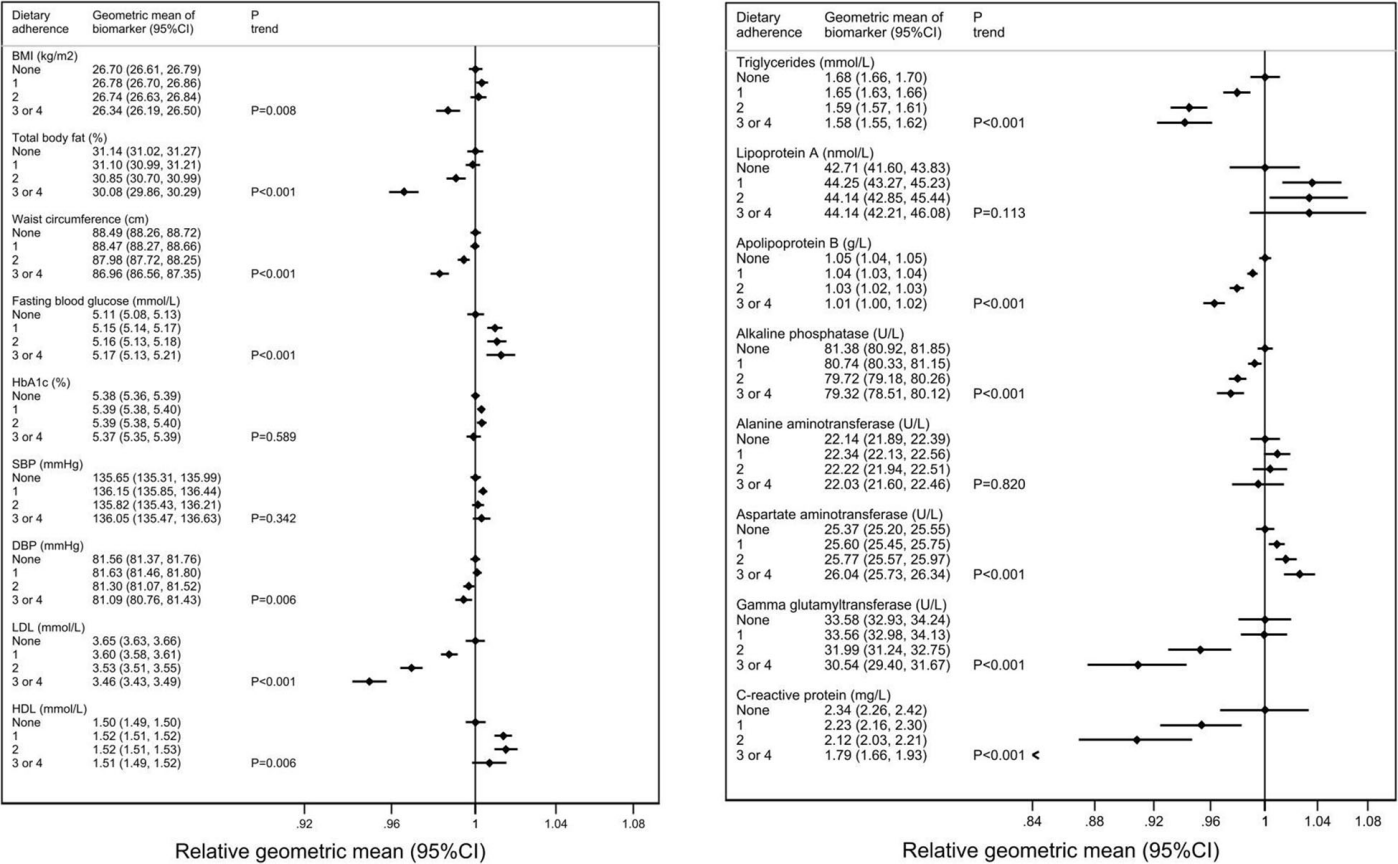
Associations between adherence to total dietary recommendations (WHO) and baseline cardiometabolic risk factors (*n* = 32,728). Abbreviations: BMI (body mass index), HbA1c (glycated haemoglobin), SBP (systolic blood pressure), DBP (diastolic blood pressure), LDL (low-density lipoprotein) cholesterol, HDL (high-density lipoprotein) cholesterol, and CI (confidence intervals). Adjusted means, 95%CI, and p-trends were estimated through multivariable logistic regression. Models included age, sex, ethnicity (Whites, others, unknown), region (England, Scotland, Wales), Townsend index of deprivation (quintiles 1–5 or unknown, with lower scores representing greater affluence), education group (vocational qualifications [NVQ, HND, HNC], any school degree [A-level, AS-level, O-level, GCSE, CSE], higher degree [college, university, of professional degree/qualification], none of the above, unknown), smoking status (never, previous, current, unknown), physical activity (continuous, total MET-hours/week), alcohol consumption (none, occasional < 1 unit/week, moderate 1–14 units/week, heavy > 14 units/week, unknown), menopausal status (yes, no, not applicable [men]), and log-transformed total daily energy as covariates

Cross-sectional analyses in a sub-sample of participants (*n* = 32,728) showed significant trends (*P*_trend_ < 0.001) between adherence to dietary recommendations and lower total body fat (1.06% difference in geometric mean between lowest and highest quintiles of adherence), waist circumference (1.53 cm), LDL cholesterol (0.19 mmol/L), triglycerides (0.10 mmol/L), apolipoprotein B (0.04 g/L), alkaline phosphatase (2.06 U/L), gamma glutamyltransferase (3.04 U/L), and hs-CRP (0.55 mg/L), but significantly higher mean values for blood glucose (0.06 mmol/L; *P*_trend_< 0.001) and aspartate aminotransferase (0.67 U/L) (Fig. 2).

## Discussion

In this large cohort of middle-aged British adults, only a third of participants met two or more of the dietary recommendations for saturated fat, free sugars, dietary fibre, or fruit and vegetables, and less than a tenth achieved three or four WHO recommendations. Adherence to a greater number of dietary recommendations was associated with a lower risk of all-cause and fatal CVD mortality. These associations were not clearly explained by any single recommendation, suggesting a cumulative effect. In cross-sectional analyses, there were some associations with established risk factors for cardiometabolic risk, including total body fat, waist circumference, LDL cholesterol, triglycerides, apolipoprotein B, alkaline phosphatase, gamma glutamyltransferase, and hs-CRP.

Our findings are consistent with a recent study using data from UK cohorts (including UK Biobank) looking at adherence to food-based recommendations based on the Eatwell plate (compared to adherence to macronutrient recommendations in our study) [4, 21]. These analyses showed that only a third of participants adhered to five food recommendations and less than 0.1% of their sample adhered to all nine of the food recommendations they examined [21]. Our study based on nutrient recommendations also supports previous research showing that greater adherence to food-based recommendations or healthy food indices is associated with lower all-cause mortality [22–24]. For example, in one study, participants in the highest quintile of the Healthy Eating Index–2015 had an 18% lower risk of all-cause mortality compared to those in the lowest quintile [23]. We similarly observed an estimated 21% reduction in all-cause mortality with increasing total adherence to nutrient-based recommendations.

We found no evidence of a trend between adherence to dietary recommendations and lower incidence of CVD incidence or mortality, after adjustment for confounders, though adherence to 3–4 recommendations was associated with lower CVD mortality. Other analyses of the overall healthfulness of the diet, as assessed by healthy dietary patterns or scores, are somewhat mixed, though systematic reviews and meta-analyses of observational and experimental studies generally support greater adherence to a variety of healthy eating patterns as protective against both CVD incidence and mortality [25, 26]. More recent longitudinal cohort studies have also shown that greater adherence to various healthy eating indices and dietary patterns was associated with a lower risk of incident and fatal CVD [23, 27]. Our cross-sectional analyses of the association between adherence to dietary recommendations and cardiometabolic risk factors were mixed, with few clinically relevant differences. However, careful interpretation is needed for these analyses which included only a subsample of people in whom biomarkers and dietary intakes were measured at the same time point. Previous cross-sectional studies have generally found that those with higher adherence to dietary guideline indices had a lower prevalence of metabolic risk factors [28–30].

Our study is strengthened by the large sample of participants with detailed measurements of dietary intake and linkage to medical records for outcome ascertainment which can minimize loss of follow-up. We also conducted sensitivity analyses to confirm the robustness of the findings while adjusting for a number of covariates that could act as confounders in the examined associations. However, our study is vulnerable to potential measurement error related to self-reporting of dietary intake. First, as the Oxford WebQ collected dietary intake over the previous 24 h, it may not be representative of usual intake. However, we included all participants who provided at least two dietary assessments to try to approximate usual intakes and reduce random error related to day-to-day variability. Our estimates may have also been affected by a systematic error related to underreporting of dietary intake. This error may have been introduced if participants forgot to report their dietary intake or deliberately under-reported specific foods and beverages. Dietary salt intake is an important nutrient for cardiovascular disease prevention which was not included in the present study due to the lack of reliable estimates of salt intake from the WebQ. Most current dietary guidelines would usually include fibre recommendations in terms of (AOAC); however, dietary fibre in the UK Biobank is an estimate of non-starch polysaccharides, which could have underestimated the percentage of participants meeting the fibre recommendation. In addition, previous analyses have suggested that participants completing more dietary assessments tended to be older and more educated compared to the general population of the UK Biobank [13], which may have affected the representativeness of our sample. Finally, not all measurements used in our cross-sectional analyses (i.e. dietary records) were taken at the same time point. As cardiometabolic variables were measured at baseline, we also do not have data on if, or how, these may have changed from the time that diet was measured to event incidence. The classification of the exposure into binary groups (meeting vs not meeting recommendations) could have further reduced the precision of our estimates compared to other methods (e.g. dietary scores).

## Conclusions

Our study provides observational evidence in a contemporary UK cohort that meeting dietary recommendations for saturated fats, sugar, fibre, and fruits and vegetables is associated with lower premature mortality. However, very few participants in this population achieved high adherence to these dietary recommendations. Motivating and supporting more people to adhere to dietary guidelines could help increase healthy life expectancy [31, 32].

## Supplementary Information

**Additional file 1.** Variables used, participant flow chart, prospective associations for individual dietary recommendations, and sensitivity analyses

## Data Availability

The datasets generated/and or analysed in the current study will be made available for bona fide researchers who apply to use the UK Biobank data set by registering and applying at http://www.ukbiobank.ac.uk/register-apply.

## Abbreviations

BMI: Body mass index
CIs: Confidence intervals
CVD: Cardiovascular disease
EI: Energy intake
EERs: Estimated energy requirements
HbA1c: Glycated haemoglobin
HDL: High-density lipoprotein
HRs: Hazard ratios
Hs-CRP: High-sensitivity C-reactive protein
ICD-10: International classification of diseases
LDL: Low-density lipoprotein
MET: Metabolic equivalent of task
NCDs: Non-communicable diseases
UK: United Kingdom
WHO: World health organization
