# Supplementary material for: Adherence to international dietary recommendations in association with all-cause mortality and fatal and non-fatal cardiovascular disease risk: a prospective analysis of UK Biobank participants
Source: BMC Med. 2021 Jun 23;19:134. doi: 10.1186/s12916-021-02011-7 (PMC8220774; doi:10.1186/s12916-021-02011-7)
Supplement: Supplementary file 1 — Additional file 1. Variables used, participant flow chart, prospective associations for individual dietary recommendations, and sensitivity analyses [file 12916_2021_2011_MOESM1_ESM.docx]

**SUPPLEMENTARY MATERIAL**

[Supplementary Table S1. Derivation of variables used in analysis from the UK Biobank study 2](#_Toc72172532)

[Supplementary Figure S1. Participant flow chart of the study 5](#_Toc72172533)

[Supplementary Figure S2. Associations between dietary adherence and all-cause mortality, total CVD, and fatal CVD risks (*n*=115 051) 6](#_Toc72172534)

[Supplementary Table S2. Associations between dietary adherence and all-cause mortality, total CVD, and fatal CVD risks, excluding people who had the event within two years after completing their last 24-h online dietary assessment (*n*=113 619) 7](#_Toc72172535)

[Supplementary Table S3. Associations between dietary adherence and all-cause mortality, total CVD, and fatal CVD risks, including participants who completed 3+, 4+ or 5+ dietary questionnaires 8](#_Toc72172536)

[Supplementary Table S4. Associations between dietary adherence and all-cause mortality, total CVD, and fatal CVD risks, by subgroups of sex, age, body mass index, smoking and presence of risk factors 9](#_Toc72172537)

### **Supplementary Table S1. Derivation of variables used in analysis from the UK Biobank study**

| Variables | Categories used in analysis | UK Biobank variable; Source (question ID) |
| --- | --- | --- |
| *Health outcomes* | | |
| BMI | Continuous, kg/m^2^ | Body mass index (BMI); BMI value constructed from height and weight measured during the initial assessment centre visit (ID: 21001)^‡^; |
| Total body fat | Continuous, % | Body fat percentage; Body composition estimated by bioimpedance, (ID: 23099)^‡^ |
| Waist circumference | Continuous, cm | Waist circumference; Tape measure (ID: 48)^‡^ |
| Blood glucose | Continuous, mmol/L | Glucose; Measured by hexokinase analysis on a Beckman Coulter AU5800 (ID: 30740)^#^ |
| HbA1c | Continuous, mmol/mol | Glycated haemoglobin (HbA1c); Measured by HPLC analysis on a Bio-Rad VARIANT II Turbo (ID: 30750)^#^ |
| Triglycerides | Continuous, mmol/L | Triglycerides; Measured by GPO-POD analysis on a Beckman Coulter AU5800 (ID: 30870)^#^ |
| LDL-c | Continuous, mmol/L | LDL direct; Measured by enzymatic protective selection analysis on a Beckman Coulter AU5800 (ID: 30780)^#^ |
| HDL-c | Continuous, mmol/L | HDL cholesterol; Measured by enzyme immunoinhibition analysis on a Beckman Coulter AU5800 (ID: 30760)^#^ |
| Lipoprotein A | Continuous, nmol/L | Lipoprotein A; Measured by immunoturbidimetric analysis on a Randox AU5800 (ID: 30790)^#^ |
| Apolipoprotein B | Continuous, g/L | Apolipoprotein B; Measured by immunoturbidimetric analysis on a Beckman Coulter AU5800 (ID: 30640)^#^ |
| hsCRP | Continuous, mg/L | C-reactive protein; Measured by immunoturbidimetric – high sensitivity analysis on a Beckman Coulter AU5800 (ID: 30710)^#^ |
| Alkaline phosphatase | Continuous, U/L | Alkaline phosphatase; Measured by AMP(IFCC) analysis on a Beckman Coulter AU5800 (ID: 30610)^#^ |
| Alanine aminotransferase | Continuous, U/L | Alanine aminotransferase; Measured by IFCC analysis on a Beckman Coulter AU5800 (ID: 30620)^#^ |
| Aspartate aminotransferase | Continuous, U/L | Aspartate aminotransferase; Measured by IFCC analysis on a Beckman Coulter AU5800 (ID: 30650)^#^ |
| Gamma glutamyltransferase | Continuous, U/L | Gamma glutamyltransferase; Measured by IFCC analysis on a Beckman Coulter AU5800 (ID: 30730)^#^ |
| Systolic blood pressure | Continuous, mmHg | Systolic blood pressure, automated reading / manual reading; Range returned by the Omron device is 0-255. A manual sphygmometer was used if the standard automated device could not be employed (ID: 4080 / 93)^‡^ |
| Diastolic blood pressure | Continuous, mmHg | Diastolic blood pressure, automated reading / manual reading; Range returned by the Omron device is 0-255. A manual sphygmometer was used if the standard automated device could not be employed (ID: 4079 / 94)^‡^ |
| Total CVD | No  Yes | Hospital admission data: ICD-10: coronary heart disease (CHD; I20-I25), congestive heart failure or cardiomyopathy (CHF; I50, I50.1, 150.9, I11.0, I13.0, I13.2, I42, I43.1), and total stroke (I60-I64) |
| Fatal CVD | No  Yes | Hospital admission data: ICD-10: coronary heart disease (CHD; I20-I25), congestive heart failure or cardiomyopathy (CHF; I50, I50.1, 150.9, I11.0, I13.0, I13.2, I42, I43.1), and total stroke (I60-I64) |
| All-cause mortality | No  Yes | Death registry data linked to the UK Biobank (ID: 40001 / 40002) |
| *Sociodemographic characteristics* | | |
| Sex | Men  Women | Sex; Acquired from central NHS registry at recruitment, but in some cases self-reported by the participant (ID: 31)* |
| Age | Continuous, years | Age at recruitment; Derived variable based on date of birth and date of attending an initial assessment centre and refers to the age of the participant on the day they attended an initial assessment centre, truncated to whole year (ID: 21022)* |
| Ethnicity | Whites  Others | Ethnic background; Amalgam of sequential branching questions asked during the initial assessment centre visit as part of the touchscreen questionnaire (ID: 21000)* |
| Townsend index | Continuous, quintiles *(high index indicates most deprivation)* | Townsend deprivation index at recruitment; Calculated immediately prior to participant joining UK Biobank based on the preceding national census output areas in which their postcode is located (ID: 189)* |
| Education | Vocational qualifications *(NVQ, HND or HNC)*  Any school degree *(A levels, AS levels, O levels, GCSEs or CSEs)*  Higher degree *(college or university degree, or professional qualifications)*  None of the above | Qualifications; ACE touchscreen question “Which of the following qualifications do you have? (You can select more than one)” (ID: 6138)^†^ |
| *Behavioural risk factors* | | |
| Alcohol | Occasional *(<1 unit/week)*  Moderate *(1 – 14 units/week)*  Heavy *(>14 units/week)*  None | Alcohol intake frequency; ACE touchscreen question “About how often do you drink alcohol?” (ID: 1558) |
| Smoking status | Never  Current  Previous | Smoking status; question asked during the initial assessment centre visit as part of the touchscreen questionnaire (ID: 20116)^†^ |
| Physical activity (Total MET hours) | Continuous (hours/week) | Number of days/week of vigorous physical activity 10+ minutes; ACE touchscreen question “In a typical WEEK, how many days did you do 10 minutes or more of vigorous physical activity? (These are activities that make you sweat or breathe hard such as fast cycling, aerobics, heavy lifting)” (ID: 904)^†^  Duration of vigorous activity; ACE touchscreen question “How many minutes did you usually spend doing vigorous activities on a typical DAY?” (ID: 914)^†^  Number of days/week of moderate physical activity 10+ minutes; ACE touchscreen question “In a typical WEEK, on how many days did you do 10 minutes or more of moderate physical activities like carrying light loads, cycling at normal pace? (Do not include walking)” (ID: 884)^†^;  Duration of moderate activity; ACE touchscreen question “How many minutes did you usually spend doing moderate activities on a typical DAY?” (ID: 894)^†^  Number of days/week walked for 10+ minutes; ACE touchscreen question “In a typical WEEK, on how many days did you walk for at least 10 minutes at a time? (Including walking that you do at work, travelling to and from work, and for sport or leisure)” (ID: 864)^†^  Duration of walks; ACE touchscreen question “How many minutes did you usually spend walking on a typical DAY?” (ID: 874)^†^ |
| Total energy intake | Continuous, kcal/day | Calculated as the total energy intake per day across 2 or more dietary assessements (Oxford WebQ) |
| *Medical conditions* | | |
| Body mass index | Underweight (<18.5 kg/m^2^)  Healthy weight (18.5 to <25 kg/m^2^)  Overweight (25 to <30 kg/m^2^)  Obese (≥30 kg/m^2^) | BMI value constructed from height and weight measured during the initial assessment centre visit (ID: 21001)^‡^; Body composition estimated by impedance measurement (ID: 23104)^‡^ |
| Menopause | No  Yes *(if responded to questions saying they have experienced menopause)* | Had menopause (women only); ACE touscreen question “Have you had your meopause (periods stopped)?” (ID: 2724)^†^ |
| Hypertension | No  Yes *(taking blood pressure medication or diagnosed with hypertension by a physician; that is, had an SBP >140 mmHg and DBP >90 mmHg)* | Vascular/heart problems diagnosed by doctor (high blood pressure is one response); ACE touchscreen question “Has a doctor ever told you that you have had any of the following conditions? (You can select more than one answer)” (ID: 6150)^†^  Systolic blood pressure, automated reading / manual reading (ID: 4080 / 93)^‡^  Diastolic blood pressure, automated reading / manual reading (ID: 4079 / 94)^‡^  Medication for cholesterol, blood pressure, or diabetes (men); ACE touscreen question “Do you regularly take any of the following medications? (You can select more than one answer)” (ID: 6177)^†^  Medication for cholesterol, blood pressure, diabetes, or take exogenous hormones (women); ACE touchscreen question “Do you regularly take any of the following medications? (You can select more than one answer)” (ID: 6153)^†^ |
| Diabetes | No  Yes *(taking medication for diabetes or HbA1c ≥6.5% or diagnosed by a physician)* | Diabetes diagnosed by doctor; ACE touchscreen question “Has a doctor ever told you that you have diabetes?” (ID: 2443)^†^  Medication for cholesterol, blood pressure, or diabetes (men); ACE touchscreen question “Do you regularly take any of the following medications? (You can select more than one answer)” (ID: 6177)^†^  Medication for cholesterol, blood pressure, diabetes, or taking exogenous hormones (women); ACE touchscreen question “Do you regularly take any of the following medications? (You can select more than one answer)” (ID: 6153)^†^  Glycated haemoglobin (HbA1c); Measured by HPLC analysis on a Bio-Rad VARIANT II Turbo (ID: 30750)^#^ |
| High Cholesterol | No  Yes *(taking cholesterol-lowering medication or LDL cholesterol >3 mmol/L or total cholesterol >5 mmol/L)* | Medication for cholesterol, blood pressure, or diabetes (men); ACE touchscreen question “Do you regularly take any of the following medications? (You can select more than one answer)” (ID: 6177)^†^  Medication for cholesterol, blood pressure, diabetes, or taking exogenous hormones (women); ACE touchscreen question “Do you regularly take any of the following medications? (You can select more than one answer)” (ID: 6153)^†^  Cholesterol; Measured by CHO-POD analysis on a Beckman Coulter AU5800 (ID: 30690)^#^  HDL cholesterol; Measured by enzyme immunoinhibition analysis on a Beckman Coulter AU5800 (ID: 30760)^#^ |
| Note: *Recruitment questions, ^†^Touchscreen questions, ^‡^Physical measurements, ^§^24-h online dietary assessment questionnaire, ^#^biological samples | | |

### **Supplementary Figure S1. Participant flow chart of the study**

502 536 participants recruited between 2006 to 2010

**115 051** participants, including:

**7 067** Total CVD cases

**886** Fatal CVD cases

**3 794** All-cause mortality case

**Exclusion criteria**

1. People without any validated 24-h online dietary assessment (*n*=291 492)
2. People with CVD events that occurred before baseline assessment or before completing their last dietary questionnaire (*n*=47 409)
3. People without 2 or more validated 24-h online dietary assessment data (*n*=1635)
4. Pregnancy (*n*=175)
5. Implausible energy intakes (under-reporters, *n*=1987, over-reporters, *n*=224),
6. BMI ≤18.5 (*n*=5437)
7. Missing fruit and vegetable intake (*n*=951)
8. Missing physical activity (*n*=1961)

### **Supplementary Figure S2. Associations between dietary adherence and all-cause mortality, total CVD, and fatal CVD risks (*n*=115 051)**

Abbreviations: SFA (saturated fatty acids), F&V (fruits and vegetables), CVD (cardiovascular disease), HR (hazard ratio), and CI (confidence intervals). Adjusted HRs, 95%CI, and p-values were estimated through multivariable Cox-proportional hazards models with mutual adjustment for all the dietary recommendations. Models included age as the underlying timescale, were stratified by sex, and adjusted for ethnicity (whites, others, unknown), region (England, Scotland, Wales), Townsend index of deprivation (quintiles 1-5 or unknown, with lower scores representing greater affluence), education group (vocational qualifications [NVQ, HND, HNC], any school degree [A-level, AS-level, O-level, GCSE, CSE], higher degree [college, university, of professional degree/qualification], none of the above, unknown), smoking status (never, previous, current, unknown), physical activity (continuous, total MET-hours/week), alcohol consumption (none, occasional <1 unit/week, moderate 1-14 units/week, heavy >14 units/week, unknown), menopausal status (yes, no, not applicable [men]), and log-transformed total daily energy.

### **Supplementary Table S2. Associations between dietary adherence and all-cause mortality, total CVD, and fatal CVD risks, excluding people who had the event within two years after completing their last 24-h online dietary assessment (*n*=113 619)**

|  | **All-cause mortality** | | | **Total CVD** | | | **Fatal CVD** | | |
| --- | --- | --- | --- | --- | --- | --- | --- | --- | --- |
|  | HR | 95% CI | | HR | 95% CI | | HR | 95% CI | |
| *Total adherence* |  |  |  |  |  |  |  |  |  |
| 0 | 1·00 | 0·94 | 1·06 | 1·00 | 0·95 | 1·05 | 1·00 | 0·88 | 1·14 |
| 1 | 0·96 | 0·91 | 1·01 | 1·05 | 1·01 | 1·09 | 0·94 | 0·83 | 1·05 |
| 2 | 0·92 | 0·86 | 0·99 | 1·06 | 1·00 | 1·12 | 1·04 | 0·90 | 1·21 |
| 3 or 4 | 0·80 | 0·72 | 0·90 | 0·98 | 0·90 | 1·07 | 0·75 | 0·58 | 0·98 |
| *Exposures* |  |  |  |  |  |  |  |  |  |
| ≤10% energy from SFA | 0·98 | 0·91 | 1·06 | 1·04 | 0·98 | 1·11 | 0·93 | 0·78 | 1·11 |
| ≥25g fibre | 0·94 | 0·84 | 1·06 | 0·96 | 0·88 | 1·05 | 0·84 | 0·66 | 1·08 |
| ≤10% energy from sugar | 0·95 | 0·88 | 1·02 | 1·01 | 0·96 | 1·07 | 1·07 | 0·92 | 1·25 |
| ≥5 servings F&V | 0·91 | 0·84 | 0·98 | 0·98 | 0·93 | 1·04 | 0·93 | 0·79 | 1·09 |

Abbreviations: SFA (saturated fatty acids), F&V (fruits and vegetables), CVD (cardiovascular disease), HR (hazard ratio), and CI (confidence intervals).

### **Supplementary Table S3. Associations between dietary adherence and all-cause mortality, total CVD, and fatal CVD risks, including participants who completed 3+, 4+ or 5+ dietary questionnaires**

| *Total adherence* | **All-cause mortality** | | | **Total CVD** | | | **Fatal CVD** | | |
| --- | --- | --- | --- | --- | --- | --- | --- | --- | --- |
|  | HR | 95% CI | | HR | 95% CI | | HR | 95% CI | |
| Three WebQs completed  n=71,659 |  |  | |  |  | |  |  | |
| 0 | 1.00 | 0.93 | 1.08 | 1.00 | 0.95 | 1.06 | 1.00 | 0.86 | 1.16 |
| 1 | 1.00 | 0.94 | 1.07 | 1.00 | 0.95 | 1.05 | 1.02 | 0.90 | 1.17 |
| 2 | 0.92 | 0.84 | 1.00 | 1.00 | 0.94 | 1.07 | 0.95 | 0.79 | 1.14 |
| 3 or 4 | 0.77 | 0.67 | 0.90 | 0.98 | 0.89 | 1.08 | 0.72 | 0.52 | 1.00 |
| Four WebQs completed  n=33,079 |  |  | |  |  | |  |  | |
| 0 | 1.00 | 0.90 | 1.11 | 1.00 | 0.92 | 1.08 | 1.00 | 0.81 | 1.24 |
| 1 | 1.00 | 0.91 | 1.10 | 1.04 | 0.97 | 1.11 | 0.99 | 0.82 | 1.20 |
| 2 | 0.87 | 0.76 | 1.00 | 1.00 | 0.91 | 1.10 | 0.91 | 0.69 | 1.20 |
| 3 or 4 | 0.78 | 0.63 | 0.97 | 0.97 | 0.83 | 1.12 | 0.73 | 0.45 | 1.17 |
| Five WebQs completed  n=5,294 |  |  | |  |  | |  |  | |
| 0 | 1.00 | 0.75 | 1.34 | 1.00 | 0.83 | 1.21 | 1.00 | 0.58 | 1.73 |
| 1 | 1.09 | 0.84 | 1.42 | 1.14 | 0.96 | 1.35 | 0.99 | 0.59 | 1.66 |
| 2 | 1.06 | 0.75 | 1.48 | 0.95 | 0.75 | 1.20 | 1.26 | 0.69 | 2.31 |
| 3 or 4 | 0.96 | 0.57 | 1.63 | 1.25 | 0.92 | 1.71 | 2.00 | 0.99 | 4.07 |

Abbreviations: CVD (cardiovascular disease), HR (hazard ratio), and CI (confidence intervals). Adjusted HRs, 95%CI, and p-values were estimated through multivariable Cox-proportional hazards models. Models included age as the underlying timescale, were stratified by sex, and adjusted for ethnicity (whites, others, unknown), region (England, Scotland, Wales), Townsend index of deprivation (quintiles 1-5 or unknown, with lower scores representing greater affluence), education group (vocational qualifications [NVQ, HND, HNC], any school degree [A-level, AS-level, O-level, GCSE, CSE], higher degree [college, university, of professional degree/qualification], none of the above, unknown), smoking status (never, previous, current, unknown), physical activity (continuous, total MET-hours/week), alcohol consumption (none, occasional <1 unit/week, moderate 1-14 units/week, heavy >14 units/week, unknown), menopausal status (yes, no, not applicable [men]), and log-transformed total daily energy.

### **Supplementary Table S4. Associations between dietary adherence and all-cause mortality, total CVD, and fatal CVD risks, by subgroups of sex, age, body mass index, smoking and presence of risk factors**

| *Total adherence* | **All-cause mortality** | | | | **Total CVD** | | | | **Fatal CVD** | | | |
| --- | --- | --- | --- | --- | --- | --- | --- | --- | --- | --- | --- | --- |
|  | Cases | HR | 95% CI | | Cases | HR | 95% CI | | Cases | HR | 95% CI | |
| **Women** |  |  |  | |  |  |  | |  |  |  | |
| 0 | 511 | 1.00 | 0.92 | 1.09 | 730 | 1.00 | 0.93 | 1.08 | 95 | 1.00 | 0.81 | 1.23 |
| 1 | 647 | 0.89 | 0.82 | 0.96 | 1029 | 0.99 | 0.93 | 1.05 | 98 | 0.74 | 0.61 | 0.91 |
| 2 | 420 | 0.91 | 0.83 | 1.00 | 646 | 0.96 | 0.89 | 1.04 | 75 | 0.91 | 0.72 | 1.14 |
| 3 or 4 | 158 | 0.75 | 0.64 | 0.87 | 286 | 0.91 | 0.81 | 1.02 | 26 | 0.67 | 0.45 | 0.98 |
| **Men** |  |  |  |  |  |  |  |  |  |  |  |  |
| 0 | 697 | 1.00 | 0.93 | 1.08 | 1416 | 1.00 | 0.95 | 1.05 | 191 | 1.00 | 0.87 | 1.15 |
| 1 | 820 | 1.02 | 0.96 | 1.10 | 1679 | 1.01 | 0.96 | 1.06 | 231 | 1.05 | 0.92 | 1.19 |
| 2 | 390 | 0.90 | 0.81 | 0.99 | 923 | 1.02 | 0.95 | 1.08 | 127 | 1.05 | 0.88 | 1.25 |
| 3 or 4 | 151 | 0.83 | 0.71 | 0.98 | 358 | 0.94 | 0.85 | 1.04 | 43 | 0.84 | 0.62 | 1.14 |
| **Age group <60 y** |  |  |  |  |  |  |  |  |  |  |  |  |
| 0 | 423 | 1.00 | 0.91 | 1.10 | 830 | 1.00 | 0.93 | 1.07 | 90 | 1.00 | 0.81 | 1.23 |
| 1 | 519 | 0.96 | 0.88 | 1.05 | 1037 | 1.00 | 0.94 | 1.06 | 90 | 0.81 | 0.66 | 1.00 |
| 2 | 286 | 0.95 | 0.84 | 1.06 | 555 | 0.96 | 0.88 | 1.04 | 67 | 1.09 | 0.85 | 1.38 |
| 3 or 4 | 108 | 0.85 | 0.71 | 1.03 | 228 | 0.94 | 0.83 | 1.07 | 14 | 0.54 | 0.32 | 0.91 |
| **Age group ≥60 y** |  |  |  |  |  |  |  |  |  |  |  |  |
| 0 | 785 | 1.00 | 0.93 | 1.07 | 1316 | 1.00 | 0.95 | 1.06 | 196 | 1.00 | 0.87 | 1.15 |
| 1 | 948 | 0.96 | 0.90 | 1.02 | 1671 | 1.01 | 0.96 | 1.06 | 239 | 1.00 | 0.88 | 1.14 |
| 2 | 524 | 0.89 | 0.82 | 0.97 | 1014 | 1.01 | 0.95 | 1.08 | 135 | 0.96 | 0.81 | 1.14 |
| 3 or 4 | 201 | 0.77 | 0.67 | 0.88 | 416 | 0.92 | 0.84 | 1.02 | 55 | 0.89 | 0.68 | 1.16 |
| **BMI 18-25** |  |  |  |  |  |  |  |  |  |  |  |  |
| 0 | 408 | 1.00 | 0.91 | 1.10 | 606 | 1.00 | 0.92 | 1.08 | 73 | 1.00 | 0.79 | 1.26 |
| 1 | 466 | 0.92 | 0.84 | 1.01 | 748 | 0.99 | 0.92 | 1.07 | 81 | 0.91 | 0.74 | 1.14 |
| 2 | 278 | 0.93 | 0.83 | 1.05 | 447 | 1.00 | 0.91 | 1.10 | 59 | 1.14 | 0.88 | 1.48 |
| 3 or 4 | 111 | 0.76 | 0.63 | 0.92 | 201 | 0.92 | 0.80 | 1.06 | 20 | 0.79 | 0.51 | 1.22 |
| **BMI 25-30** |  |  |  |  |  |  |  |  |  |  |  |  |
| 0 | 530 | 1.00 | 0.92 | 1.09 | 979 | 1.00 | 0.94 | 1.07 | 130 | 1.00 | 0.84 | 1.19 |
| 1 | 601 | 0.91 | 0.84 | 0.99 | 1205 | 1.00 | 0.95 | 1.06 | 135 | 0.86 | 0.73 | 1.02 |
| 2 | 299 | 0.79 | 0.70 | 0.89 | 666 | 0.97 | 0.90 | 1.04 | 79 | 0.90 | 0.72 | 1.12 |
| 3 or 4 | 124 | 0.78 | 0.66 | 0.93 | 290 | 1.00 | 0.89 | 1.12 | 28 | 0.76 | 0.52 | 1.10 |
| **BMI ≥30** |  |  |  |  |  |  |  |  |  |  |  |  |
| 0 | 270 | 1.00 | 0.89 | 1.13 | 561 | 1.00 | 0.92 | 1.09 | 83 | 1.00 | 0.80 | 1.24 |
| 1 | 400 | 1.09 | 0.99 | 1.20 | 755 | 0.98 | 0.91 | 1.05 | 113 | 1.01 | 0.84 | 1.21 |
| 2 | 233 | 1.05 | 0.92 | 1.19 | 456 | 0.98 | 0.89 | 1.07 | 64 | 0.94 | 0.73 | 1.20 |
| 3 or 4 | 74 | 0.85 | 0.67 | 1.06 | 153 | 0.83 | 0.71 | 0.98 | 21 | 0.79 | 0.51 | 1.22 |
| **Never smoker** |  |  |  |  |  |  |  |  |  |  |  |  |
| 0 | 573 | 1.00 | 0.92 | 1.09 | 1105 | 1.00 | 0.94 | 1.06 | 128 | 1.00 | 0.84 | 1.19 |
| 1 | 651 | 0.93 | 0.87 | 1.01 | 1320 | 0.99 | 0.94 | 1.05 | 143 | 0.94 | 0.80 | 1.11 |
| 2 | 369 | 0.90 | 0.81 | 0.99 | 765 | 0.98 | 0.91 | 1.05 | 85 | 0.95 | 0.77 | 1.17 |
| 3 or 4 | 146 | 0.82 | 0.70 | 0.96 | 332 | 0.97 | 0.87 | 1.08 | 32 | 0.82 | 0.58 | 1.16 |
| **Former smoker** |  |  |  |  |  |  |  |  |  |  |  |  |
| 0 | 468 | 1.00 | 0.91 | 1.10 | 814 | 1.00 | 0.93 | 1.07 | 115 | 1.00 | 0.83 | 1.20 |
| 1 | 625 | 0.97 | 0.90 | 1.05 | 1140 | 1.02 | 0.96 | 1.08 | 147 | 0.97 | 0.83 | 1.14 |
| 2 | 362 | 0.92 | 0.83 | 1.02 | 712 | 1.05 | 0.97 | 1.13 | 98 | 1.09 | 0.89 | 1.32 |
| 3 or 4 | 152 | 0.84 | 0.72 | 0.99 | 293 | 0.95 | 0.84 | 1.06 | 37 | 0.91 | 0.66 | 1.26 |
| **Current smoker** |  |  |  |  |  |  |  |  |  |  |  |  |
| 0 | 166 | 1.00 | 0.85 | 1.17 | 223 | 1.00 | 0.87 | 1.15 | 43 | 1.00 | 0.88 | 1.14 |
| 1 | 190 | 1.04 | 0.91 | 1.20 | 247 | 1.02 | 0.90 | 1.15 | 39 | 0.84 | 0.56 | 1.25 |
| 2 | 76 | 0.98 | 0.78 | 1.23 | 89 | 0.85 | 0.69 | 1.05 | 17 | 0.87 | 0.51 | 1.50 |
| 3 or 4 | 11 | 0.39 | 0.21 | 0.70 | 19 | 0.49 | 0.32 | 0.78 | 0 | n/a | n/a | n/a |
| **Presence of risk factors No** |  |  |  |  |  |  |  |  |  |  |  |  |
| 0 | 78 | 1.00 | 0.80 | 1.25 | 85 | 1.00 | 0.81 | 1.24 | 14 | 1.00 | 0.58 | 1.71 |
| 1 | 88 | 0.91 | 0.74 | 1.13 | 105 | 1.01 | 0.84 | 1.23 | 10 | 0.62 | 0.33 | 1.16 |
| 2 | 49 | 0.90 | 0.68 | 1.20 | 75 | 1.24 | 0.99 | 1.56 | 7 | 0.85 | 0.40 | 1.80 |
| 3 or 4 | 29 | 1.04 | 0.72 | 1.51 | 29 | 0.94 | 0.65 | 1.36 | 3 | 0.71 | 0.23 | 2.23 |
| **Presence of risk factors Yes** |  |  |  |  |  |  |  |  |  |  |  |  |
| 0 | 1130 | 1.00 | 0.94 | 1.06 | 2061 | 1.00 | 0.96 | 1.04 | 272 | 1.00 | 0.89 | 1.13 |
| 1 | 1379 | 0.97 | 0.92 | 1.02 | 2603 | 1.00 | 0.96 | 1.04 | 319 | 0.96 | 0.86 | 1.07 |
| 2 | 761 | 0.91 | 0.85 | 0.98 | 1494 | 0.98 | 0.94 | 1.04 | 195 | 1.01 | 0.88 | 1.16 |
| 3 or 4 | 280 | 0.77 | 0.69 | 0.87 | 615 | 0.93 | 0.86 | 1.01 | 66 | 0.79 | 0.62 | 1.00 |

Abbreviations: CVD (cardiovascular disease), HR (hazard ratio), and CI (confidence intervals). Adjusted HRs, 95%CI, and p-values were estimated through multivariable Cox-proportional hazards models. Models included age as the underlying timescale, were adjusted for age (except for the model stratified by age group), sex (except for the model stratified by sex), ethnicity (whites, others, unknown), region (England, Scotland, Wales), Townsend index of deprivation (quintiles 1-5 or unknown, with lower scores representing greater affluence), education group (vocational qualifications [NVQ, HND, HNC], any school degree [A-level, AS-level, O-level, GCSE, CSE], higher degree [college, university, of professional degree/qualification], none of the above, unknown), smoking status (never, previous, current, unknown), physical activity (continuous, total MET-hours/week), alcohol consumption (none, occasional <1 unit/week, moderate 1-14 units/week, heavy >14 units/week, unknown), menopausal status (yes, no, not applicable [men]), and log-transformed total daily energy. Presence of risk factors is defined as having a diagnosis of hypertension, diabetes, or high cholesterol.
